# Late Mortality, Subsequent Malignant Neoplasms and Hospitalisations in Long-Term Survivors of Adolescent and Young Adult Hematological Cancers

**DOI:** 10.3389/fonc.2022.823115

**Published:** 2022-02-25

**Authors:** Annalisa Trama, Claudia Vener, Paolo Lasalvia, Alice Bernasconi, the Ada Working Group, Manuel Zorzi

**Affiliations:** ^1^ Evaluative Epidemiology Unit, Department of Research, Fondazione IRCCS Istituto Nazionale dei Tumori, Milan, Italy; ^2^ Oncology and Haemato-Oncology Department, University of Milan, Milan, Italy

**Keywords:** long-term outcomes, adolescents and young adults (AYAs), hematological cancers, cancer survivors, population-based cohort

## Abstract

**Background:**

Increased success in the treatment of hematological cancers contributed to the increase of 5-year survival for most adolescent and young adults (AYAs) with these tumours. However, as 5-year survival increased, it became clear that AYA long-term survivors were at increased risk for severe late effects. Moreover, limited information on long-term cancer impact is available for AYAs, since most studies focused on children and adolescents. We aimed to assess various long-term outcomes on AYA survivors of hematological cancers.

**Methods:**

We selected patients diagnosed with a first primary hematological cancer between 1997 and 2006, in the Italian nationwide population-based cohort of AYA cancer survivors (i.e. alive at least 5 years after cancer diagnosis). Long-term outcomes of interest were: second malignant neoplasms (SMNs), hospitalizations and overall mortality. We calculated standardized incidence ratios (SIRs), standardized hospitalization rate ratios (SHRs) and standardized mortality rate ratios (SMRs). To study morbidity patterns over time, we modeled observed incidence rates by fitting flexible parametric models for nonlinear patterns and we used linear regression for linear patterns.

**Results:**

The study cohort included 5,042 AYA hematological cancer survivors of which 1,237 and 3,805 had a leukaemia and lymphoma diagnosis, respectively. AYA survivors were at substantially increased risk for SMN (SIR=2.1; 95%CI=1.7; 2.6), hospitalisation (SHR=1.5; 95%CI=1.5; 1.6), and mortality (SMR=1.4; 95%CI=1.2; 1.6) with differences between leukaemia and lymphoma survivors. The highest excess risks of hospitalisations were for infectious diseases, respiratory diseases, and diseases of blood and blood-forming organs. The morbidity pattern differs over time by morbidity type.

**Conclusions:**

Our results support the need for strict follow-up plans for survivors, and call for further study to better personalised follow-up plans for AYA cancer survivors.

## Introduction

Hematological tumours are common cancers in adolescents and young adults (15-39 years at cancer diagnosis; AYAs), especially in males and younger AYAs (1). Survival for hematological cancers (acute lymphoid leukemias, acute myeloid leukemias, Hodgkin’s lymphomas, non-Hodgkin lymphomas), is significantly worse in AYAs than in children but it is good and continuously improving (2), thus an increasing number of young people are becoming long-term cancer survivors.

Long-term outcomes in AYA cancer survivors are not well understood and are largely extrapolated from survivors of childhood cancer. However, AYAs have different cancer types from children and adults, and the biology of AYA cancers is distinct. AYAs may handle treatment differently and have different late effects. Furthermore, adolescence and young adulthood is a challenging developmental phase. The problem is that studies have focused mainly on childhood cancer survivors’ late effects. Because cancer in AYAs is so different to cancer in children, findings derived from childhood studies cannot be extrapolated to AYA cancer survivors (3). Furthermore, the available studies on the long-term impact of cancer on AYAs have focused mainly on a single long-term outcome (4–8) or a single tumour (9–12).

Considering the dearth of information on AYAs, it is becoming very important to study late effects in AYA cancer survivors to optimize management that will reduce number and impact of adverse effects.

Against this background, we aim to provide a comprehensive assessment of diverse long-term health outcomes (i.e. subsequent malignant neoplasms (SMNs), all-cause mortality, and hospitalisations) on survivors of all and recently diagnosed hematological cancers. Taking advantage of the Italian nationwide cohort of AYA cancer survivors, we will consider the AYA cancer patient population as a whole (15-39 years). This is the first Italian nation-wide cohort of AYA cancer survivors which takes advantage of large population-based cancer registries (CRs) and, through large-scale record linkage techniques, with health database, death registries, and hospital registries, provides accurate follow-up information on AYA cancer survivors (13).

## Materials and Methods

The AYA cancer survivor cohort has been described elsewhere (13). Briefly, it is a retrospective incident-based cohort derived from CRs. Each CR identified patients with a first cancer diagnosis between the ages of 15 and 39 years during the entire incidence period covered, linking them to all their SMNs, hospital discharge records (HDRs), and mortality data. AYA cancer survivors were subsequently defined as those patients alive at least 5 years after the first cancer diagnosis. CRs contributed to the cohort with different incidence periods, depending on the year of establishment. As of September 2021, about 30 CRs contributed to the cohort with 67,692 AYA cancer survivors diagnosed between 1976 and 2013.

This paper focuses on AYA hematological cancer survivors. Hematological tumours were defined according to the International Classification of Childhood Cancer, Third Edition ICCC-3 (14): Group I “Leukaemia, myeloproliferative diseases, and Myelodysplastic diseases” and Group II “Lymphomas and reticuloendothelial neoplasms”. We divided lymphomas into Hodgkin lymphomas (HLs) and non-Hodgkin lymphomas (NHLs) (except Burkitt lymphoma), as ICCC-3 Group IIa and Group IIb, respectively, and other lymphomas (**Supplementary Table 1**). Furthermore, leukemias were divided according to the histology codes of the International Classification of Diseases for Oncology, Third Edition (ICD-O-3), into acute leukemias (ALs), chronic leukemias (CLs), and other leukemias (**Supplementary Table 1**). Only tumours with malignant behaviour were included in the analysis (ICD-O-3 behaviour=3).

Outcomes of interest were: SMNs, hospitalisations (used as a proxy of chronic comorbid conditions), and overall mortality. An SMN was defined as a malignant neoplasm of any site with different morphology from the first primary tumour, according to recommended multiple primary cancer coding (15). The aim of these rules is to distinguish recurrences or progressive disease from multiple primary cancers. SMNs were provided by CRs. Hospital admissions were grouped into 10 main diagnostic groups, converting ICD-8 and ICD-10 codes to ICD-9 CM codes, as in Rubjerg et al. (16): Infectious and parasitic diseases (001–139), Endocrine diseases and other related diseases (240-279), Diseases of blood and blood-forming organs (280-289), Diseases of nervous system and sense organs (320-389), Diseases of circulatory system (390-459), Diseases of respiratory system (460-519), Diseases of digestive organs (520-579), Diseases of urinary system and genital organs (580-629), Diseases of the skin and subcutaneous tissue (680-709), and Diseases of bone, joint, and soft tissue (710-739). Hospitalisations were retrieved from HDRs. Mortality was retrieved from the regional mortality registries.

**Figure 1:**
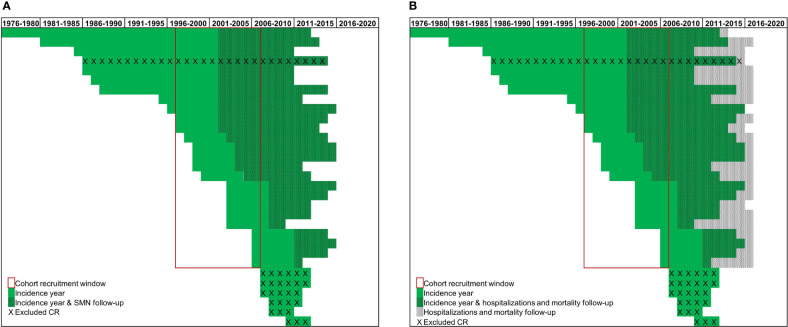
Incidence and available follow-up **(A)** for Second Malignant Neoplasms (SMNs) and **(B)** for hospitalizations and mortality, over time by cancer registries (CR) (green lines).

To maximise both the representativeness of the CRs and the follow-up time for each outcome of interest, we selected AYAs diagnosed with a first primary hematological cancer between 1997 and 2006 (**Figure 1**, cohort recruitment window). Of the 31 CRs included in the cohort, 6 CRs were excluded because cancer registration started outside the selected cohort window (i.e. from 2007 onwards) and 1 CR was excluded because the HDRs were missing. Ultimately, 24 CRs (**Figure 1**, red box) contributed to these analyses. The 24 included CRs covered about 34% of the Italian population from different geographical areas. Follow-up for cancer incidence was available for most CRs until 2012, while HDR and mortality files were available until 2016 (**Figure 1**, dotted lines).

### Statistical Analyses

The three outcomes of interest (SMNs, all-cause mortality, and hospitalisations) were analysed separately. To avoid inclusion of most acute, sub-acute, non-persistent, or treatment-induced conditions, follow-up began 5 years after the date of cancer diagnosis. For each outcome, the follow-up ended on the date of emigration, last known vital status, specific outcome of interest occurrence (SMN, cause-specific first hospitalization or death), last availability of the linked data source (incidence date entered by the CRs, HDRs or mortality registry) or the closing date (31 December 2012 for SMNs or 31 December 2016 for mortality and hospitalisations). Only the first event (SMN and specific hospital admission) was considered in the analyses.

We estimated the excess risk of SMNs, hospitalizations, and all-cause mortality in AYA hematological cancer survivors by standardized incidence ratios (SIRs), standardized hospitalization rate ratios (SHRs), and standardized mortality rate ratios (SMRs), respectively. SIRs, SHRs, and SMRs were all calculated as the ratio between observed and expected events. Observed events refer to events experienced by the cohort during the follow-up period. Expected events are events the AYA cohort would have experienced had their risk been the same as the general population. Expected events were calculated by multiplying the incidence rates of the general population, matched by gender, area of residence, attained age (5-year band), and calendar year (1-year band), with the person-year at risk accrued by the AYA survivor cohort. Expected general population rates for SMNs, hospitalizations, and mortality were calculated using complete cancer incidence data provided by the CRs, Italian nationwide HDRs (13), and ISTAT (the Italian National Institute of Statistics) mortality tables (17), respectively. We calculated 95% confidence intervals (CI) assuming a Poisson distribution.

We identified non-linear hospitalization rates over time, modelling observed incidence rates by fitting flexible parametric models (18). We used linear regression to describe linear hospital admissions over time. To visualize the excess risk over time, we graphically present observed and expected hospitalization rates over time. We calculated expected events by multiplying the incidence rates of the general population, thus precluding formal assessment of the variability of the expected rates. This is why we have simply described the differences between the curves without comparing them statistically. All analyses were performed using Stata 17.

## Results

The cohort of 5,042, 5-year AYA hematological cancer survivors had a median follow-up time of 10 years (interquartile range 8-12) for SMNs and of 13 years (interquartile range 11-15) for hospitalisations and mortality. Most 5-year AYA survivors were males (54%) and were diagnosed during adulthood (30-39 years, 55%) (**Table 1**). Leukemias were reported in 25% of AYA survivors and most leukaemia survivors had CL (60%). Among leukaemia patients, as expected, AL was more common in adolescents while CL was more common in young adults. Lymphomas were described in 75% of AYA survivors of whom 54% were HL and 40% were NHL. Among patients with lymphoma, HL were more frequent in 15-19 and 20-29 years olds whereas NHL was more frequent in young adults (30-39 years).

**Table 1:** Characteristics of adolescent and young adult hematological cancer survivor cohort, overall and by type of hematological cancer, by sex and age at diagnosis.

	Total	Sex				Age at diagnosis					
		Male	%	Female	%	15-19	%	20-29	%	30-39	%
**Overall**	5042	2702	54%	2340	46%	584	12%	1681	33%	2777	55%
Leukaemias	1237	674	25%	563	24%	117	20%	346	21%	774	28%
-Acute Leukaemias	427	229	9%	198	8%	81	14%	132	8%	214	8%
-Chronic Leukaemias	745	418	15%	327	14%	32	5%	202	12%	511	18%
-Other Leukaemias	65	27	1%	38	2%	4	1%	12	1%	49	2%
Lymphomas	3805	2028	75%	1777	76%	467	80%	1335	79%	2003	72%
-Hodgkin Lymphomas	2,048	1002	37%	1046	45%	358	61%	875	52%	815	29%
-Non-Hodgkin Lymphomas	1,533	894	33%	639	27%	84	15%	385	23%	1064	38%
-Other Lymphomas	224	132	5%	92	4%	25	4%	75	4%	124	5%

Percentages (%) are calculated as column totals except for overall (total for the row).

**Table 2** shows SIRs, SHRs, and SMRs overall, by sex and type of first hematological cancer. AYA hematological cancer survivors were at substantially increased risk for SMN (SIR=2.1; 95%CI=1.7; 2.6), hospitalisation (SHR=1.5; 95%CI=1.5; 1.6), and mortality (SMR=1.4; 95%CI=1.2; 1.6). SIR, SHR, and SMR were 2.4 (95%CI=1.7;3.3) and 1.9 (95%CI=1.4; 2.5); 1.7 (95%CI=1.6; 1.8) and 1.4 (95%CI=1.3; 1.5); 1.3 (95%CI=1.1; 1.5) and 1.7 (95%CI=1.4; 2.0) in males and females, respectively. Lymphoma survivors were at greatest risk of developing SMN, being more than two-fold higher (SIR=2.2; 95%CI=1.7; 2.8) than the age-specific and gender-specific rates. NHLs and HLs had equal SIR values, but NHLs showed higher SHRs and SMRs than HL survivors. The highest risk for leukaemia survivors was hospitalisation (SHR=1.8; 95%CI=1.6; 1.9). However, CL survivors had an 80% excess risk of developing any SMN (SIR=1.8; 95%CI=1.0; 3.2). For AL survivors, the number of observed events was too small to draw conclusions on the excess risk of SMN. No major differences in terms of SMR were observed between AYA survivors of lymphoma and leukaemia.

**Table 2:** Observed and expected numbers of events (O/E), Standardized Incidence Ratios (SIRs) of Second Malignant Neoplasms, Standardized Hospitalisation rate Ratios (SHRs) and Standardized Mortality rate Ratios (SMRs) with 95% confidence intervals (95% CI): overall and stratified by sex and primary hematological tumor.

	Subsequent Malignant Neoplasms			Hospitalisations			Mortality		
	O/E	SIR	95% CI	O/E	SHR	95% CI	O/E	SMR	95% CI
**Overall**	86/41	2.1	[1.7; 2.6]	3226/2092	1.5	[1.5; 1.6]	291/209	1.4	[1.2; 1.6]
**Sex**									
Male	39/16	2.4	[1.7; 3.3]	1786/1053	1.7	[1.6; 1.8]	179/142	1.3	[1.1; 1.5]
Female	47/25	1.9	[1.4; 2.5]	1440/1038	1.4	[1.3; 1.5]	112/67	1.7	[1.4; 2.0]
**First primary hematological tumour**									
Leukaemias (including other leukaemias)	18/11	1.7	[1.1; 2.7]	895/510	1.8	[1.6; 1.9]	78/54	1.4	[1.2; 1.8]
-Acute Leukaemias	5/3	1.5	[0.6; 3.7]	299/163	1.8	[1.6; 2.1]	31/16	2.0	[1.4; 2.8]
-Chronic Leukaemias	12/7	1.8	[1.0; 3.2]	521/322	1.6	[1.5; 1.8]	38/36	1.1	[0.8; 1.5]
Lymphomas (including other lymphomas)	68/31	2.2	[1.7; 2.8]	2331/1582	1.5	[1.4; 1.5]	213/155	1.4	[1.2; 1.6]
-Hodgkin Lymphomas	32/14	2.2	[1.6; 3.1]	1025/806	1.3	[1.2; 1.4]	82/70	1.2	[0.9; 1.4]
-Non-Hodgkin Lymphomas	32/15	2.2	[1.5; 3.1]	1167/683	1.7	[1.6; 1.8]	122/75	1.6	[1.4; 1.9]

**Figure 2:**
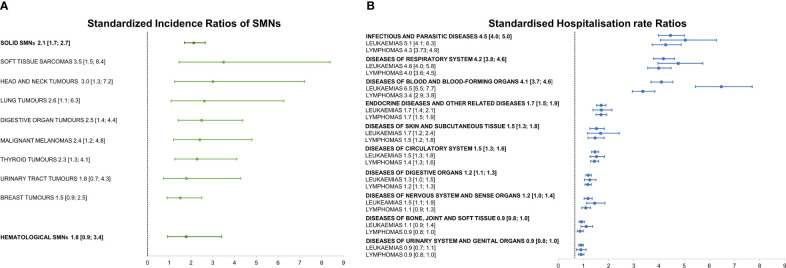
**(A)** Standardized Incidence Ratios of Second Malignant Neoplasms (SMNs) stratified as hematological and solid and by subsequent solid malignant tumor type; **(B)** Standardized Hospitalisation rate Ratios stratified by cause of hospitalisation and hematological first primary cancer, with 95% confidence intervals in brackets.

AYA cured from their hematological cancer were at high risk for solid (SIR=2.1; 95%CI=1.7; 2.7) and for hematological SMNs (SIR=1.8; 95%CI=0.9; 3.4) (**Figure 2A**). It should, however, be underlined that the hematological SIR was not statistically significant. SIRs for subsequent primary soft tissue sarcoma, melanoma, and cancers of the head and neck, lung, digestive tract, and thyroid rose significantly, but the increase did not achieve statistical significance for urinary tract and breast cancer. The highest excess risks of hospitalisation (four-fold compared to the general population not affected by a primary cancer during young adulthood) were for infectious diseases (SHR=4.5; 95%CI=4.0; 5.0), respiratory diseases (SHR=4.2; 95%CI=3.8; 4.6), and diseases of blood and blood-forming organs (SHR=4.1; 95%CI=3.7; 4.6), followed by diseases of the endocrine system, skin, circulatory system, and digestive organs (**Figure 2B**). SHRs for infectious and respiratory diseases, and diseases of blood and blood-forming organs were higher for leukaemia than for lymphoma survivors.

**Figure 3:**
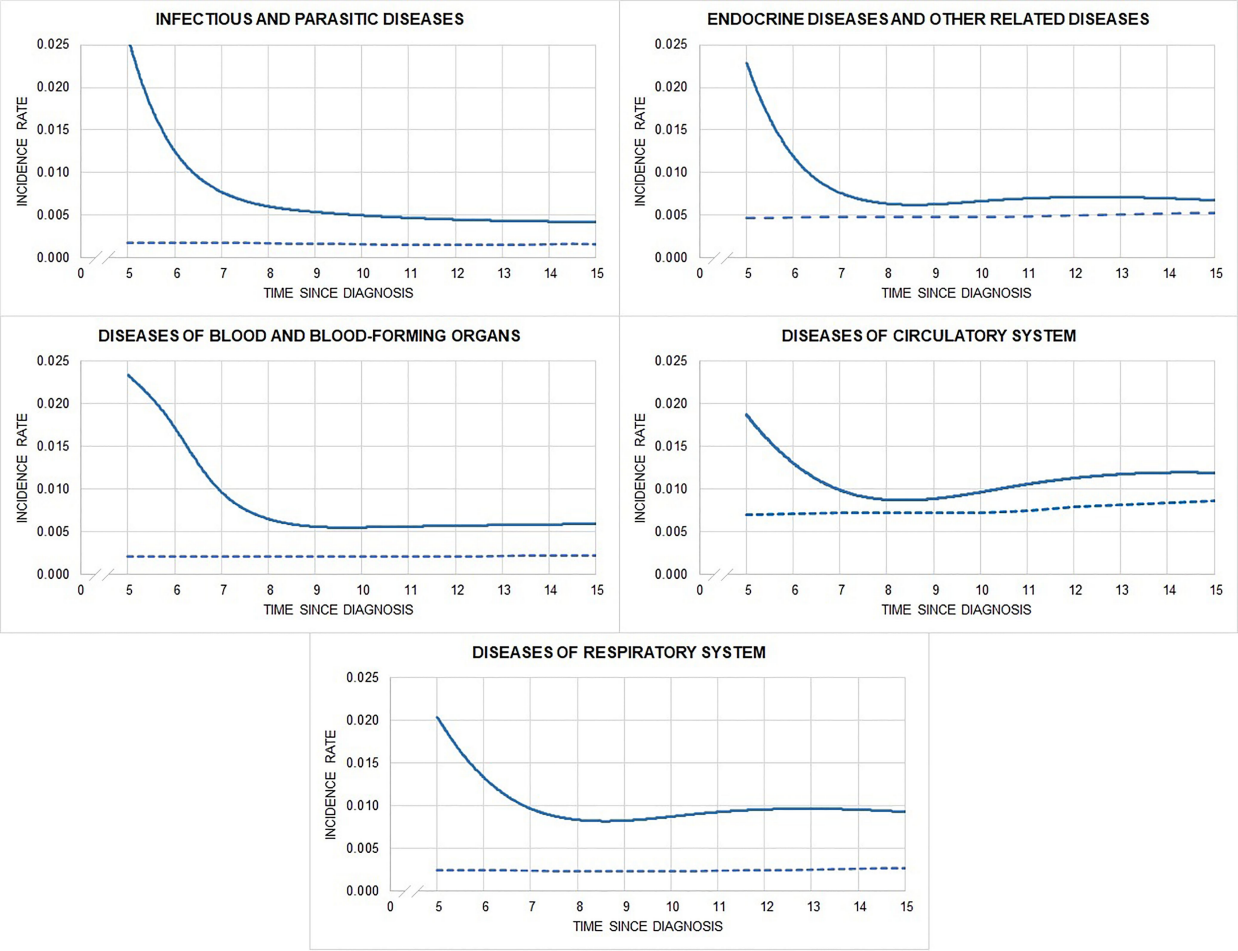
Observed (solid) and expected (dashed) hospitalisation rates by time since diagnosis and main diagnostic groups of hospitalization.

**Figure 3** shows hospitalisation rates over time from 5 years after cancer diagnosis, by main diagnostic group (only hospital admissions with a non-linear trend are reported; those with a linear trend are in **Supplementary Table 1**). The incidence of infectious, endocrine, and blood and blood-forming organ diseases was highest close to the time of cancer diagnosis, declining over time, while the incidence of circulatory and respiratory system diseases was highest close to the time of cancer diagnosis, but then decreased and increased again at year 9. The SHR of AYA hematological cancer survivors remained higher (compared to general population who did not have a primary cancer during young adulthood) up to 20 years from cancer diagnosis. For central nervous and urinary system, skin, digestive organ, and bone diseases, hospitalisation rates did not differ over time from those of the general population (**Supplementary Figure 1**).

## Discussion

AYA hematological cancer survivors were at substantially increased risk of SMNs, hospitalizations, and mortality. Previous studies have focused on late effects that are most likely to increase the risk of death. We report all the different types of late effects showing that AYA cancer survivors will face several health problems that will impact their quality of life as well as increase their risk of dying. Notably, survivors not only had an increased risk of chronic diseases but also experienced several such diseases (on average 3). This is important to properly inform AYA survivors and personalize their follow-up. Available studies are difficult to compare due to varying methods for defining risk, age groups (e.g. 15-24; 15-29 years), and primary cancer, in addition to differences in study period, follow-up, and comparison groups. In any event, our results support previous evidence of an excess risk of death (8, 9), SMNs (7, 19), and hospitalizations (16, 20–22) for AYA hematological cancer survivors compared to the general population. We report about the AYA cancer patient population as a whole (15-39 years).

Furthermore, we focused on a recent diagnosis period (1997-2006) during which improved cancer treatment, including targeted and precision therapies, should have reduced the therapeutic burden used. Our results confirmed that SMRs, SMNs, and SHRs are lower in more recent periods of diagnosis. Bhuller et al. (9) reported increased late morbidity (SIR=7.8) and mortality risk (SMR=8.8) for 442 teenage and young adult 5-year survivors of HL, diagnosed at 15–24 years of age, between 1970 and 1999. However, SMRs and SIRs were lower for survivors diagnosed in 1990-1999 compared to 1970-1979. Anderson et al. (8) observed a substantial decrease in 5-year all-cause mortality and primary cancer-specific mortality between the earliest (1975-1984) and most recent (2005-2011) diagnosis periods for several cancer types, including leukaemia, NHL, and HL. Kumar (23) reported that HL patients diagnosed between 1973 and 1986 had a 12% greater risk of developing secondary cancers (HR=1.12; 95%CI, 1.03-1.23; P = 0.01) compared with patients diagnosed between 1987 and 2000.

Our results have shown that solid tumors are the most common SMNs. We also observed that the risk of developing any SMN was higher for NHL and HL than for leukaemia survivors. Moreover, among leukaemia survivors, CLs showed higher SIRs than did ALs. These differences may be related to the natural course of the diseases, especially to the longer treatment burden for some lymphomas and CLs compared to ALs, in which treatment tends to be concentrated over a shorter time. The elevated risk of subsequent solid cancers (lung, breast, stomach, and pancreas) has been largely attributed to radiation therapy and particularly to high radiation doses. For lung cancer, the increased relative risk from smoking appeared to multiply the elevated risks from radiotherapy. We do not have data on smoking habits in our cohort. However, previous studies showed that compared with controls, survivors reported smoking tobacco at the same rate or higher rate (24–27); based on the *Behavioural Risk Factor Surveillance* Italian (*Progressi delle Aziende Sanitarie per la Salute in Italia [PASSI]*) among 18-24 and 25-34 years old, smoking prevalence was 30% and 33%, respectively (28). Furthermore, not only radiotherapy but also alkylating chemotherapy can substantially increase the risk of solid malignancies, particularly of lung, stomach, and pancreatic cancer (12), while anthracycline exposure can heighten the risk of breast cancer and other solid malignancies, including sarcoma (29). Immunosuppression, exposure to ultraviolet radiation, and genetic factors have been purported to generate a host environment conducive to the development of malignant melanoma, NHL or chronic lymphatic leukaemia (CLL) (30). Finally, chemotherapy and radiation therapy have been most closely explored as possible risk factors for therapy-related myelodysplastic syndrome and acute myeloid leukaemia (31). Unfortunately, we do not have data on treatment details.

The pattern of hospitalization is similar among hematological cancer survivors, with the highest SHRs for infectious, respiratory, and blood and blood-forming organ diseases. However, the SHR was higher for leukaemia than for lymphoma survivors. This is likely the result of intensive treatments with immunosuppressive agents more commonly used in leukemias than in other hematological disorders, for which targeted and precision therapies have been available for many years (32–36). Unfortunately, we lack data on treatment details to support and discuss our hypothesis in detail. Note should also be taken of the high SHRs for endocrine and circulatory system diseases. Treatment for HL includes irradiation to the thyroid region, which increases the risk of thyroid diseases. In addition, evidence has shown that total body irradiation performed in preparation for bone marrow transplantation results in high risks for gonadal dysfunction, thyroid dysfunction, and adrenal abnormalities (5, 37). In our cohort, the most common dysfunctions observed in the endocrine diagnostic group were thyroid diseases and diabetes. A high incidence of cardiovascular disease among patients with leukaemia and NHL has previously been found and attributed to several cancer therapies (4, 21, 38, 39). The occurrence of cardiovascular disease events has also been associated with a substantially heightened risk of death (21), suggesting that the identification and mitigation of cardiovascular disease risk factors in these high-risk populations may improve long-term patient outcomes. While we observed a high SHR for disease of the circulatory system, we were unable to assess the cause of death. However, we did find that AYA survivors who died tended to have several chronic comorbid conditions, including cardiovascular diseases.

Our study has several strengths, including the unbiased population-based approach, the reliability of Italian CR data, the longitudinal nature of the data, and cohort coverage. Our cohort covers 34% of the Italian population and includes CRs from different geographical areas of northern, central, and southern Italy. It is thus reasonably representative of areas characterized by different lifestyles, which may have a relevant impact on the chronic comorbid conditions observed. This is also the first study to systematically characterize the development of chronic comorbidities, including SMNs, among survivors of AYA cancer in Italy. Nonetheless, our study does also have some limitations. CRs do not collect data on cancer stage, treatment, or genetic information. Our outcomes are time-dependent measures hence our results directly depend on observed follow-up time. We intentionally selected CRs to maximize the follow-up, but since we are focusing on a recent period of diagnosis, follow-up of our cohort may not be sufficient to provide a comprehensive burden of long-term comorbid conditions.

To conclude, AYA hematological cancer survivors face many life transitions in terms of education, employment, social relations, relocations, and family formation. Late effects could thus have far more physical and social consequences for AYAs than for older adults. Our study, assessing multiple types of morbidities, has highlighted that survivors of adolescent and young adult hematological cancers face persistent risks (at least 20 years from diagnosis) for a broad range of diseases underscoring the need for strict evidence-based follow-up plans for survivors, designed to increase the likelihood of early detection and ultimately prevent chronic treatment-induced conditions. Our findings have also shown that the morbidity pattern differs over time by morbidity type. The incidence of some diseases (infectious, endocrine, and blood and blood-forming organ diseases) was highest close to the time of cancer diagnosis and declined over time, while the incidence of others (circulatory and respiratory system diseases) was highest close to the time of cancer diagnosis, but then decreased and increased again at year 9. Having information on when patients are at greatest risk is very important in defining personalized follow-up strategies that minimize the burden of follow-up exams.

## Ada Working Group

Manuel Zorzi, Anita Andreano, Paolo Contiero, Gianfranco Manneschi, Fabio Falcini, Marine Castaing, Rosa Angela Filiberti, Cinzia Gasparotti, Claudia Cirilli, Rosalba Amodio, Isabella Bisceglia, Silvia Iacovacci, Maria Francesca Vitale, Fabrizio Stracci, Maria Adalgisa Gentilini, Rosario Tumino, Simona Carone, Giuseppe Sampietro, Anna Melcarne, Luciana Gatti, Lorenza Boschetti, Mariangela Corti, Magda Rognoni, Enzo Coviello, Maria Teresa Pesce, Giancarlo D’Orsi, Anna Clara Fanetti, Lucia De Lorenzis, Giuseppa Candela, Fabio Savoia, Cristiana Pascucci, Maurizio Castelli, Cinzia Storchi.

## Data Availability Statement

The original contributions presented in the study are included in the article/**Supplementary Material**. Further inquiries can be directed to the corresponding author.

## Author Contributions

AT, CV, PL, and AB contributed to writing-original draft and writing-review and editing. AB and PL contributed also to formal analysis, investigation, software and methodology. AT contributed also to conceptualization, project administration and methodology. Ada working group contributed to data collection. All authors approved the submitted version.

## Funding

This work was supported by 5x1000 Funds—2013, Italian Ministry of Health and European Commission (Work Programme 2017, Grant Agreement number 801520 HP-JA-2017 ‘‘Innovative Partnership for Action Against Cancer’’).

## Conflict of Interest

The authors declare that the research was conducted in the absence of any commercial or financial relationships that could be construed as a potential conflict of interest.

## Publisher’s Note

All claims expressed in this article are solely those of the authors and do not necessarily represent those of their affiliated organizations, or those of the publisher, the editors and the reviewers. Any product that may be evaluated in this article, or claim that may be made by its manufacturer, is not guaranteed or endorsed by the publisher.
