# Supplementary material for: Late Mortality, Subsequent Malignant Neoplasms and Hospitalisations in Long-Term Survivors of Adolescent and Young Adult Hematological Cancers
Source: Front Oncol. 2022 Feb 25;12:823115. doi: 10.3389/fonc.2022.823115 (PMC8913709; doi:10.3389/fonc.2022.823115)

Supplementary Material

# Supplementary Figures and Tables

## Supplementary Tables

**Supplementary Table 1**: Leukaemias and lymphomas by histology code grouping

| **Group** | **ICDO3M** | **Description** |
| --- | --- | --- |
| Acute leukaemias | 98013 | Acute leukaemia, NOS |
|  | 98053 | Acute biphenotypic leukaemia |
|  | 98263 | Burkitt cell leukaemia |
|  | 98273 | Adult T-cell leukaemia/lymphoma (HTLV-1 pos.) |
|  | 98323 | Prolymphocytic leukaemia, NOS |
|  | 98333 | Prolymphocytic leukaemia, B-cell type |
|  | 98343 | Prolymphocytic leukaemia, T-cell type |
|  | 98353 | Precursor cell lymphoblastic leukaemia, NOS |
|  | 98363 | Precursor B-cell lymphoblastic leukaemia |
|  | 98373 | T lymphoblastic leukaemia/lymphoma |
|  | 98403 | Acute myeloid leukaemia, M6 type |
|  | 98613 | Acute myeloid leukaemia |
|  | 98663 | Acute promyelocytic leuk., t(15;17)(q22;q11-12) |
|  | 98673 | Acute myelomonocytic leukaemia |
|  | 98703 | Acute basophilic leukaemia |
|  | 98713 | Ac. myelomonocytic leuk. w abn. mar. eosinophils |
|  | 98723 | Acute myeloid leukaemia, minimal differentiation |
|  | 98733 | Acute myeloid leukaemia without maturation |
|  | 98743 | Acute myeloid leukaemia with maturation |
|  | 98913 | Acute monocytic leukaemia |
|  | 98953 | Acute myeloid leuk. with multilineage dysplasia |
|  | 98963 | Acute myeloid leukaemia, t(8;21)(q22;q22) |
|  | 98973 | Acute myeloid leukaemia, 11q23 abnormalities |
|  | 99103 | Acute megakaryoblastic leukaemia |
|  | 99203 | Therapy-related acute myeloid leukaemia, NOS |
|  | 99303 | Myeloid sarcoma |
|  | 99313 | Acute panmyelosis with myelofibrosis |
|  | 99483 | Aggressive NK-cell leukaemia |
|  | 99833 | Refractory anemia with excess blasts |
|  | 99843 | Refract. anemia with excess blasts in transformation |
|  | 99873 | Therapy-related myelodysplastic syndrome, NOS |
| Chronic leukaemias | 98233 | Chronic lymphocytic leukaemia/small lymphocytic lymphoma |
|  | 98313 | T-cell large granular lymphocytic leukaemia |
|  | 98633 | Chronic myeloid leukaemia, NOS |
|  | 98753 | Chronic myelogenous leukaemia, BCR/ABL positive |
|  | 98763 | Atypical chronic myeloid leuk., BCR/ABL negative |
|  | 99403 | Hairy cell leukaemia |
|  | 99453 | Chronic myelomonocytic leukaemia, NOS |
|  | 99463 | Juvenile myelomonocytic leukaemia |
|  | 99503 | Polycythemia vera |
|  | 99603 | Chronic myeloproliferative disease, NOS |
|  | 99613 | Myelosclerosis with myeloid metaplasia |
|  | 99623 | Essential thrombocythemia |
|  | 99633 | Chronic neutrophilic leukaemia |
|  | 99643 | Hypereosinophilic syndrome |
|  | 99753 | Myelodysplastic/Myeloproliferative neoplasm, unclassifiable |
|  | 99803 | Refractory anemia |
|  | 99853 | Refractory cytopenia with multilineage dysplasia |
|  | 99863 | Myelodysplastic syndr. with 5q deletion syndrome |
| Other leukaemias | 98003 | Leukaemia, NOS |
|  | 98203 | Lymphoid leukaemia, NOS |
|  | 98603 | Myeloid leukaemia, NOS |
|  | 99893 | Myelodysplastic syndrome, NOS |
| Hodgkin lymphomas | 96503 | Hodgkin lymphoma, NOS |
|  | 96513 | Hodgkin lymphoma, lymphocyte-rich |
|  | 96523 | Hodgkin lymphoma, mixed cellularity, NOS |
|  | 96533 | Hodgkin lymphoma, lymphocytic deplet., NOS |
|  | 96543 | Hodgkin lymph., lymphocyt. deplet., diffuse fibrosis |
|  | 96553 | Hodgkin lymphoma, lymphocyt. deplet., reticular |
|  | 96593 | Hodgkin lymph., nodular lymphocyte predom. |
|  | 96613 | Hodgkin granuloma [obs] |
|  | 96623 | Hodgkin sarcoma [obs] |
|  | 96633 | Hodgkin lymphoma, nodular sclerosis, NOS |
|  | 96643 | Hodgkin lymphoma, nod. scler., cellular phase |
|  | 96653 | Hodgkin lymphoma, nod. scler., grade 1 |
|  | 96673 | Hodgkin lymphoma, nod. scler., grade 2 |
| non-Hodgkin lymphomas | 95913 | Malignant lymphoma, non-Hodgkin |
|  | 96703 | ML, small B lymphocytic, NOS |
|  | 96713 | ML, lymphoplasmacytic |
|  | 96733 | Mantle cell lymphoma |
|  | 96753 | ML, mixed sm. and lg. cell, diffuse |
|  | 96783 | Primary effusion lymphoma |
|  | 96793 | Mediastinal large B-cell lymphoma |
|  | 96803 | ML, large B-cell, diffuse |
|  | 96843 | ML, large B-cell, diffuse, immunoblastic, NOS |
|  | 96893 | Splenic marginal zone B-cell lymphoma |
|  | 96903 | Follicular lymphoma, NOS |
|  | 96913 | Follicular lymphoma, grade 2 |
|  | 96953 | Follicular lymphoma, grade 1 |
|  | 96983 | Follicular lymphoma, grade 3 |
|  | 96993 | Marginal zone B-cell lymphoma, NOS |
|  | 97003 | Mycosis fungoides |
|  | 97013 | Sezary syndrome |
|  | 97023 | Mature T-cell lymphoma, NOS |
|  | 97053 | Angioimmunoblastic T-cell lymphoma |
|  | 97083 | Subcutaneous panniculitis-like T-cell lymphoma |
|  | 97093 | Cutaneous T-cell lymphoma, NOS |
|  | 97143 | Anaplastic large cell lymphoma, T-cell and Null cell type |
|  | 97163 | Hepatosplenic gamma-delta cell lymphoma |
|  | 97173 | Intestinal T-cell lymphoma |
|  | 97183 | Primary cutan. CD30+ T-cell lymphoprolif. disorder |
|  | 97193 | NK/T-cell lymphoma, nasal and nasal-type |
|  | 97273 | Precursor cell lymphoblastic lymphoma, NOS |
|  | 97283 | Precursor B-cell lymphoblastic lymphoma |
|  | 97293 | Precursor T-cell lymphoblastic lymphoma |
|  | 97313 | Plasmacytoma, NOS |
|  | 97323 | Multiple myeloma |
|  | 97333 | Plasma cell leukaemia |
|  | 97343 | Plasmacytoma, extramedullary |
|  | 97603 | Immunoproliferative disease, NOS |
|  | 97613 | Waldenstrom macroglobulinemia |
|  | 97623 | Heavy chain disease, NOS |
|  | 97643 | Immunoproliferative small intestinal disease |
| Other lymphomas | 95903 | Malignant lymphoma, NOS |
|  | 95963 | Composite Hodgkin and non-Hodgkin lymphoma |
|  | 96873 | Burkitt lymphoma, NOS |
|  | 97403 | Mast cell sarcoma |
|  | 97413 | Malignant mastocytosis |
|  | 97423 | Mast cell leukaemia |
|  | 97503 | Malignant histiocytosis |
|  | 97543 | Langerhans cell histiocytosis, disseminated |
|  | 97553 | Histiocytic sarcoma |
|  | 97563 | Langerhans cell sarcoma |
|  | 97573 | Interdigitating dendritic cell sarcoma |
|  | 97583 | Follicular dendritic cell sarcoma |

## Supplementary Figures

**Supplementary Figure 1:** Observed (solid) and expected (dashed) hospitalisation rates by time since diagnosis and main diagnostic groups of hospitalisation


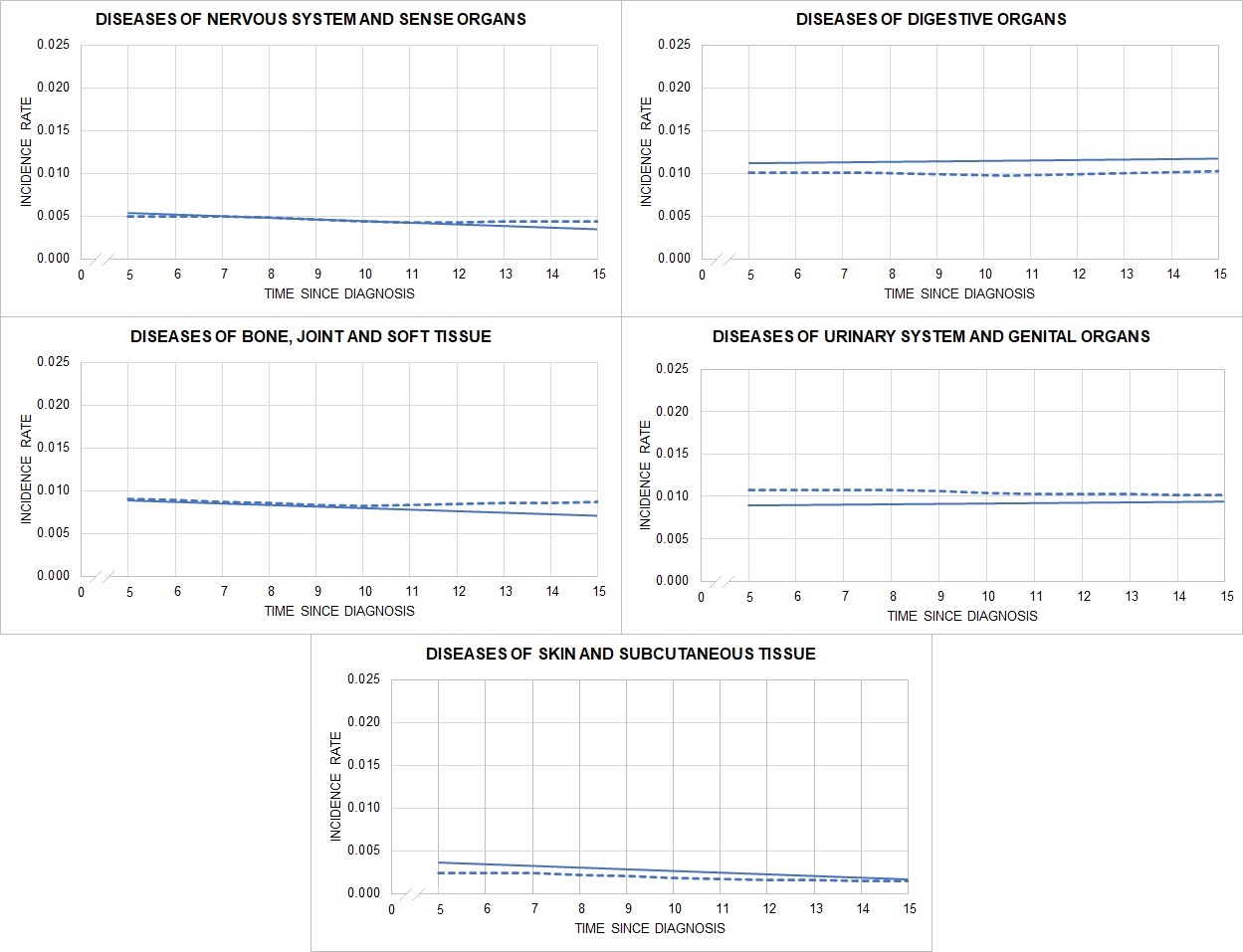

Supplement: Supplementary file 1 [file DataSheet_1.docx]
